# Viral suppression among pregnant adolescents and women living with HIV in rural KwaZulu-Natal, South Africa: a cross sectional study to assess progress towards UNAIDS indicators and Implications for HIV Epidemic Control

**DOI:** 10.1186/s12978-022-01419-5

**Published:** 2022-05-12

**Authors:** Nonzwakazi P. Ntombela, Ayesha B. M. Kharsany, Adenike Soogun, Nonhlanhla Yende-Zuma, Cheryl Baxter, Hans-Peter Kohler, Lyle R. McKinnon

**Affiliations:** 1 Centre for the AIDS Programme of Research in South Africa, University of KwaZulu-Natal, Congella, South Africa; 2 2nd Floor, Doris Duke Medical Research Institute, School of Laboratory Medicine and Medical Sciences, Nelson R Mandela School of Medicine, CAPRISA, University of KwaZulu-Natal, Private Bag 7, Congella, 4013 Durban South Africa; 3 Population Studies Center, University of Pennsylvania, New York, USA; 4 Department of Medical Microbiology and Infectious Diseases, University of Manitoba, Winnipeg, Canada; 5 Current Affiliation for Dr Cheryl Baxter, Centre for Epidemic Response and Innovation (CERI), School for Data Science and Computational Thinking, Stellenbosch University, Stellenbosch, South Africa

**Keywords:** Pregnant adolescents and women, HIV, HIV testing, ART, Viral suppression

## Abstract

**Background:**

South Africa has made significant progress in scaling up antiretroviral therapy (ART) to achieve the aspirational goal of HIV epidemic control. The aim of this study was to determine the prevalence of HIV, assess progress towards each of the Joint United Nations Programme on HIV/AIDS (UNAIDS) indicators and determine factors associated with achieving viral suppression among pregnant adolescents and women living with HIV in rural KwaZulu-Natal, South Africa.

**Methods:**

Pregnant adolescents and women, 12 years and older seeking antenatal care at six primary health care clinics were enrolled in a cross-sectional study. Following written informed consent, structured questionnaires were administered, and finger-prick blood samples were collected for HIV antibody testing and viral load measurement. Viral suppression was defined as HIV viral load of < 400 copies per mL.

**Results:**

Between Dec 2016 and March 2017, among the 546 enrolled participants, data for 545 were analysed. The overall HIV prevalence was 40.2% [95% Confidence Interval (CI) 36.1–44.3]. Age-stratified prevalence increased from 22.1% (95% CI, 15.9–30.0) in the 14–19 year age group to 63.9% (95% CI, 55.1–71.9) among women ≥ 30 years (*Χ*^2^ trend *P* < 0.0001). Of the HIV positive participants, 84.5% (95% CI, 79.0–88.8) knew their HIV positive status, 98.3% (95% CI 95.1–99.4) who knew their status were on ART, and of those on ART, 95.9% (95% CI 91.8–98.0) were virally suppressed. Among all HIV-positives 90.8% (95% CI, 86.3–94.0) had achieved viral suppression, whilst those in the 14–19 year age group were least likely to be virally suppressed at 82.8% (95% CI 65.5–92.4) compared to those in the older age groups. Married women compared to those unmarried were more likely to have achieved viral suppression (PRR) of 1.11 (95% CI 1.05–1.18), *P* < 0.001.

**Conclusions:**

The proportion of HIV positive pregnant women achieving viral suppression was encouraging though far short of the target towards achieving epidemic control. Importantly, adolescent pregnant women were less likely to know their HIV status and to achieve viral suppression, underscoring the public health implications of sustained risk of HIV transmission. Thus, greater effort and strong social support are essential to improve HIV knowledge of status and care continuum towards the goal to achieving HIV epidemic control.

**Plain language summary:**

To “fast-track” the response to achieve HIV epidemic control and end the AIDS epidemic, the Joint United Nations Programme on HIV/AIDS (UNAIDS) set ambitious HIV testing and treatment targets for people living with HIV. Meeting these targets through scaling up testing for HIV, initiating and sustaining antiretroviral therapy (ART) to maintain viral suppression provides both therapeutic and preventive benefits with the potential to reduce HIV transmission. Viral suppression among pregnant adolescents and women living with HIV is crucial for the prevention of mother-to-child transmission of HIV including onward transmission to sexual partners. As a public health approach, in South Africa all pregnant women are offered routine HIV testing and immediate initiation of lifelong ART irrespective of CD4 cell count. It is, therefore, important to ascertain progress towards reaching the targets. The proportion of HIV positive pregnant adolescents and women achieving viral suppression was encouraging though far short of the target towards achieving epidemic control. Importantly, pregnant adolescents were less likely to know their HIV status and to achieve viral suppression, underscoring the public health implications of sustained risk of HIV transmission. Thus, greater effort and strong social support are essential to improve HIV knowledge of status and care continuum towards the goal to achieving HIV epidemic control.

**Supplementary Information:**

The online version contains supplementary material available at 10.1186/s12978-022-01419-5.

## Background

In 2014, the Joint United Nations Programme on HIV/AIDS (UNAIDS) set ambitious HIV testing and treatment targets to “Fast-Track” the response to help end the AIDS epidemic [1]. These targets specified that 90% of all people living with HIV (PLHIV) should know their status; 90% of all people diagnosed with HIV should receive sustained antiretroviral therapy (ART) and 90% of all people receiving ART should achieve HIV viral suppression with a goal of reducing AIDS-related morbidity and mortality and the number new HIV infections [2]. Country-level commitment and resources to meet these indicators had been prioritized as the strategy was expected to provide both therapeutic and preventive benefits [1], with the potential to prevent onward transmission of HIV [3] and at a population level to reduce annual HIV incidence to less than 1% [1, 4]. Despite the efforts to meet these targets, the unequal distribution of ART, AIDS related mortality and the slow reduction in HIV incidence, many high HIV burden countries missed achieving these targets [5, 6]. The Global AIDS Strategy of 2021–2026 is yet another bold approach that has prioritized sexual reproductive health and rights for adolescents and women living with HIV, urging countries to overcome barriers preventing progress and raised the targets to 95–95–95 to be met by 2025 towards the control of HIV epidemic by the year 2030 [7].

In South Africa, the substantial scale-up of ART has resulted in reduced numbers of HIV-related deaths [8], nevertheless nationally, HIV prevalence and incidence remains persistently high [9] and prevalence among pregnant women was reported to be 30.7% [10] with the province of KwaZulu-Natal the worst affected with a HIV prevalence of 41.1% compared to the Western Cape with a prevalence of 15.9% [10]. In a region where heterosexual sex is the major route of HIV transmission, women of reproductive age are severely affected with an increased potential of mother to child transmission (MTCT) of HIV during the period of pregnancy, childbirth, or breastfeeding [10]. Thus, viral suppression is crucial for the prevention of mother-to-child transmission (PMTCT) of HIV [11, 12] including transmission to sexual partners. As a public health approach, all pregnant women are offered routine HIV testing and immediate initiation of lifelong ART irrespective of CD4 cell count [12, 13]. Furthermore, multiple programmes and interventions provide guidelines on designing, implementing and sustaining adolescent friendly services and models of HIV care and to support families and communities to improve health and well-being of adolescents [14, 15]. In the context of high HIV burden settings, pregnant women provide a reasonable population [16], to assess the effectiveness of HIV prevention and treatment programs [17] and monitor the evolving HIV epidemic trajectories [18, 19]. Therefore, the aim of this study was to determine the prevalence of HIV, assess progress towards each of the UNAIDS indicators and determine factors associated with achieving viral suppression among pregnant women living with HIV in rural KwaZulu-Natal, South Africa.

## Methods

### Study design, setting and population

This cross-sectional study was undertaken across six public sector primary health care (PHC) clinics in the rural uMgungundlovu district in KwaZulu-Natal, between December 2016 to March 2017. Whilst this community has access to basic utility services such as water, sanitation, and electricity, the area is poor and is characterized by high rates of poverty, unemployment, and HIV [3]. The PHC clinics deliver basic health care services at no cost including HIV testing services with pre- and post-test counseling with linkage to care and ART initiation.

To strengthen HIV testing services, policies and procedures were implemented to align with international guidelines and recommendations such that the services were responsive to community needs and supportive of the South Africa National Strategic Plan for HIV, STIs and TB (NSP) [20, 21] and geared to the broader goal of the National Development Plan, Vision 2030 [22] and to the UNAIDS 90–90–90 indicators [23]. HIV testing services evolved to include HIV self-testing [24] and index testing [25] to strengthen routine offering of safe and ethical services for adolescents [14, 15] including for all family members and clients of people living with HIV (PLHIV); thus the policies aimed to enable “fast-tracking” towards achieving the goals aligned to the indicators [26]. The sample for this study was drawn from 1700 first visit attendees, the numbers who attend clinics on average per year, resulting in a coverage of almost 30% of seropositive samples, given the high HIV prevalence observed among pregnant women [27, 28].

### Study procedures

Study information was provided to PHC clinic staff, who supported the study and guided study staff to designated clinic areas to facilitate access to and recruitment of study participants. Pregnant adolescents and women, 12 years and older attending PHC clinics for the current pregnancy were invited for study participation. Age-eligible participants were provided with study information. For those willing to participate, we obtained written informed consent in English or isiZulu for those 18 years and older. For those younger than 18 years we obtained assent and facilitated parental consent through the provision of study information and information on the South African legal framework that enables young children to access sexual reproductive health services autonomously from age 12. Furthermore, the community in the district acknowledges the high HIV burden and the CAPRISA Research Support Group facilitates research engagement for young people [27, 29]. Following consenting procedures, fingerprints were obtained with a mobile biometric scanner for identifying and preventing re-enrollment of study participants. Enrolled participants were assigned a unique study number and a corresponding barcode that linked the questionnaire data, finger prick blood samples, laboratory results and biometric fingerprints.

The standardized questionnaires were designed to obtain sociodemographic, behavioral, and clinical information including self-reported knowledge of HIV status, recency of HIV testing, linkage to PMTCT and uptake of ART. Questionnaires were pre-programed onto handheld personal digital assistant (PDA) tablets (MobenziR Researcher, Durban, South Africa) and administered by study staff. The appropriate selection options (i.e., single response, multiple response, open ended questions, drop down lists, etc.) were included to ensure that data were captured as accurately as possible. Skip patterns were incorporated to ensure that only required questions were completed whilst questions that were compulsory compelled interviewer to complete before proceeding. To maintain confidentiality, participants personal information was not included on the questionnaire. To safeguard the study data, the data were delivered through a wireless cellular connection and once transmitted the data were deleted from the PDA. Study staff were allocated user rights through secure passwords to enable administering of questionnaires.

All participants provided finger-prick blood samples into BD Microtainer® (Becton Dickinson, South Africa) blood collection tubes for laboratory measurements. HIV antibody testing was performed in a central laboratory using the fourth generation HIV enzyme BioMerieux Vironostika Uniform II Antigen/Antibody Microelisa system (BioMérieux, Marcy I’Etoile, France). Reactive samples were confirmed as positive with the HIV 1/2 Combi Roche Elecys (Roche Diagnostics, Penzberg, Germany). Any indeterminate results were resolved with ADVIA Centaur HIV Antigen/Antibody Combo (CHIV) Assay (Siemens, Tarry Town, NY, USA). HIV viral load was measured using the Roche COBAS AmpliPrep/COBAS TaqMan HIV-1 v2.0 assay (CAP/CTM HIV-1 V2.0, Roche Diagnostics, Penzberg, Germany) with a dynamic range of 20–10 million copies per mL Participants received their HIV antibody test and viral load results through clinic staff. HSV-2 antibodies were determined by the detection of human IgG class antibodies using the HerpeSelect1 HSV-2 enzyme-linked immunosorbent assay (Focus Diagnostics, Cypress, CA, USA) test [30].

### Statistical analysis

Statistical analyses were performed using SAS (SAS Institute, Cary, North Carolina) version 9.4.

Data were summarized using descriptive summary measures, expressed as proportions for categorical variables and central measures of tendency for medians with interquartile range (IQR) for continuous variables. To determine the progress towards each of the 90–90–90 indicators, the study sample was age-stratified into 14–19, 20–24, 25–29 and ≥ 30 years age groups. The Wilson score test assessed the age-specific HIV prevalence, the 90–90–90 targets by study characteristics and the corresponding 95% Confidence Interval (CI). The Cochran–Armitage Chi-square (*X*^2^) test was used to assess for linear trends in HIV prevalence. Based on self-reported data, one sample test of proportion using Wilson score test assessed the proportions and 95% CI for participants who achieved the “first 90”, “second 90” and the “third 90”. HIV viral suppression was defined as viral load < 400 copies per mL, as the potential risk for HIV viral transmission had been shown to be absent at HIV viral load of ≤ 400 copies per mL [31, 32]. All variables were tested for multicollinearity using Spearman’s rank correlation and variables that correlated were excluded from the model. Independent variables included in each of the multivariable models were age, education level, marital status, and the number of lifetime sexual partners. In evaluating each association, we fitted multivariable modified Poisson regression models to calculate prevalence risk ratio (PRR) and 95% CI. Statistical significance was assessed at 5% level for all analyses. The study followed the Strengthening the Reporting of Observational Studies in Epidemiology (STROBE) reporting guidelines [33].

## Results

### Description of study population

A total of 610 pregnant clinic attendees were approached and invited to participate in the study. Of these, 64 (10.5%) were excluded. The reasons for exclusion were refusal (n = 27, 42.2%), not returning for enrolment after routine clinic procedures (n = 26, 40.6%), illness (n = 2, 3.1%), being late for work (n = 5, 7.8%), being mentally incapable of undergoing consent (n = 1, 1.6%), not completing study procedures (n = 1, 1.6%), lack of parental consent (n = 1, 1.6%), and reason unknown (n = 1, 1.6%). Five hundred and forty-six women provided written informed consent and / or assent and were enrolled. Of these, 545 had finger prick bloods samples collected and were included in HIV prevalence measurement, whilst 539 had complete questionnaire data and included in the behavioural measurements (Additional file 1: Fig. S1).

Table 1 shows the baseline characteristics of study participants (denominators may vary due to missing data and has been indicated accordingly). The median age was 24 years (IQR: 20–28]. Just over half, 53.6% (n = 289/539) had completed high school, whilst 46.0% (n = 245/532) reported a total household income of ≤ 5000 South African Rand (ZAR) per month (ZAR15 ~ US$1) and 9.6% (n = 52/532) reported being married. About three quarters, 69.0% (n = 372/539) reported that their biological mother was alive, whilst less than half, 44.5% (n = 240/539) reported that their biological father was alive. Sexual debut had occurred at ≤ 16 years of age in 11.6% (n = 63/545), at 17–18 years in 19.6% (n = 107/545) and at > 18 years in 61.5% (n = 335/545) and 64.2% (n = 346/539) reported having two or more lifetime sex partners. Just under half, 48.2% (n = 260/537) reported the current pregnancy to be the first; 37.2% (n = 194/522) reported that their partner was circumcised; 29.5% (n = 159/539) reported ever having symptoms of sexually transmitted infections (STI) and 61.8% (n = 337/545) were positive for HSV-2 antibodies.

**Table 1:** Baseline characteristics of enrolled pregnant adolescents and women in a rural KwaZulu-Natal, South Africa

Characteristics	Study sample*			
Age (median, IQR)	24 (20–28)			
Socio-demographic characteristics				
Age group in years (n. %)				
14–19	131		24.0	
20–24	184		33.8	
25–29	108		19.8	
≥ 30	122		22.4	
Education levels (n. %)				
Completed high school	289		53.6	
Incomplete high school	250		46.4	
Household income per month (n. %)				
≤ ZAR 5000	245		46.0	
> ZAR 5000	110		20.6	
Don’t know	178		33.4	
Marital status				
Married	52		9.6	
Unmarried	487		90.4	
Family characteristics				
Biological mother alive				
Yes	372		69.0	
No	163		30.2	
Don’t know	4		0.7	
Biological father alive				
Yes	240		44.5	
No	290		53.8	
Don’t know	9		1.7	
Behavioral characteristics				
Age at first sex				
At ≤ 16 years	63		11.6	
At 17–18 years	107		19.6	
At > 18 years	335		61.5	
Refused	40		7.3	
Total number of lifetime sex partners				
1 partner	162		30.1	
2 or more partners	346		64.2	
Don’t know/Refused	31		5.8	
Total number of current sex partners (n,%)				
1 partner	520		96.5	
2 or more partners	4		0.7	
Refused	15		2.8	
Alcohol use (n,%)				
No	339		62.9	
Yes	200		37.1	
Drug use (n,%)				
No	463		88.7	
Yes	59		11.3	
HIV Testing history				
Last HIV test done (n, %)				
> 12 months	78		14.6	
≤ 12 months	456		85.4	
Clinical characteristics				
Pregnancies (n, %)				
Current only	260		48.2	
2 or more	277		51.4	
The first time that you had sex, was your partner circumcised (n.%)				
No	250		47.9	
Yes	194		37.2	
Don’t know	78		14.9	
Ever had any STI symptoms (n. %)				
Yes	159		29.5	
No	380		70.5	
HSV-2 antibodies (n, %)				
Positive	337		61.8	
Negative	208		38.2	

^*^Missing data were excluded from percentage calculation

^*^Refused and don’t know data were included in percentage calculation

Age-group 14–19 years – although the inclusion criteria was 12 year and older, we did not have any participants that were less than 14 years

IQR = Interquartile range; ZAR = South African Rand (ZAR15 ~ US$1); ART = Antiretroviral therapy; STI = sexually transmitted infections

Ever had any STI symptoms = any symptoms of abnormal vaginal discharge, burning or pain when passing urine, or presence of any genital ulcers/warts; HSV-2 = Herpes simplex virus type 2

### Prevalence of HIV

Table 2 shows the overall HIV prevalence of 40.2% [95% CI, 36.1–44.3], (n = 219/545). HIV prevalence increased by age and was 22.1% (95% CI, 15.9–30.0), (n = 29/131) in the 14–19 years age group, 29.9% (95% CI, 23.7–36.9), (n = 55/184) in the 20–24 years age group, 52.8% (95% CI, 43.4–61.9), (n = 57/108) in the 25–29 years age group and 63.9% (95% CI, 55.1–71.9), (n = 78/122) in the ≥ 30 year age group (*Χ*^2^ trend *P* < 0.001). Whilst HIV prevalence was similar across most variables, prevalence was higher among women with two or more lifetime sex partners (50.6%, n = 175/346)) and in those who refused to answer or responded with “don’t know” (67.7%, n = 21/31) compared to having one partner (13.0%, n = 21/162); women who had an HIV test done > 12 months ago (88.5%, n = 69/78) compared to ≤ 12 months (31.6%, n = 144/456); women with two or more pregnancies (51.6%, n = 143/277) compared to current pregnancy only (28.2%, n = 73/260); women whose partner was not circumcised (51.2%, n = 128/250) and don’t know of partners circumcision status (47.4%, n = 37/78) compared to knowing that partner was circumcised (21.6%, n = 42/194) and women who were HSV-2 antibody positive (57.3%, n = 193/337) compared to those testing negative (12.5%, n = 26/208), (all *P* < 0.001).

**Table 2:** HIV prevalence by baseline characteristics of pregnant adolescents and women in a rural KwaZulu-Natal, South Africa

Characteristics	HIV prevalence^a^			
	n/N		% (95% CI)	
Overall	219/545		40.2 (36.1–44.3)	
Socio-demographic characteristics				
Age group in years				
14–19	29/131		22.1 (15.9–30.0)	
20–24	55/184		29.9 (23.7–36.9)	
25–29	57/108		52.8 (43.4–61.9)	
≥ 30	78/122		63.9 (55.1–71.9)	
Education levels				
Completed high school	110/289		38.1 (32.7–43.8)	
Incomplete high school	107/250		42.8 (36.8–49.0)	
Household income per month				
≤ ZAR 5000	96/245		39.2 (33.3–45.4)	
> ZAR 5000	48/110		43.6 (34.7–53.0)	
Don’t know	71/178		39.9 (33.0–47.2)	
Marital status				
Married	26/52		50.0 (36.9–63.1)	
Unmarried	191/487		39.2 (35.0–43.6)	
Family characteristics				
Biological mother alive				
Yes	133/372		35.8 (31.1–40.7)	
No	82/163		50.3 (42.7–57.9)	
Don’t know	2/4		50.0 (15.0–85.0)	
Biological father alive				
Yes	81/240		33.8 (28.1–39.9)	
No	133/290		45.9 (40.2–51.6)	
Don’t know	3/9		33.3 (12.1–64.6)	
Behavioral characteristics				
Age at first sex				
At ≤ 16 years	12/63		19.0 (11.2–30.4)	
At 17–18 years	30/107		28.0 (20.4–37.2)	
At > 18 years	168/335		50.1 (44.8–55.5)	
Refused	9/40		22.5 (12.3–37.5)	
Total number of lifetime sex partners				
1 partner	21/162		13.0 (8.6–19.0)	
2 or more partners	175/346		50.6 (45.3–55.8)	
Don’t know/Refused	21/31		67.7 (50.1–81.4)	
Total number of current sex partners				
1partner	207/520		39.8 (35.7–44.2)	
2 or more partners	1/4		25.0 (45.6–70.0)	
Refused	9/15		60.0 (35.8–80.2)	
Alcohol use				
No	136/339		40.1 (35.0–45.4)	
Yes	81/200		40.5 (33.9–47.4)	
Drug use (n,%)				
No	187/463		40.4 (36.0–44.9)	
Yes	23/59		39.0 (27.6–51.7)	
HIV Testing history				
Last HIV test done				
> 12 months	69/78		88.5 (79.5–93.8)	
≤ 12 months	144/456		31.6 (27.5–36.0)	
Clinical characteristics				
Pregnancies				
Current only	73/260		28.1 (23.0–33.8)	
2 or more	143/277		51.6 (45.8–57.4)	
The first time that you had sex, was your partner circumcised				
No	128/250		51.2 (45.0–57.3)	
Yes	42/194		21.6 (16.4–28.0)	
Don’t know	37/78		47.4 (36.7–58.4)	
Ever had any STI symptoms				
Yes	78/159		49.1 (41.4–56.8)	
No	139/380		36.6 (31.9–41.5)	
HSV-2 antibodies				
Positive	193/337		57.3 (51.9–62.4)	
Negative	26/208		12.5 (8.7–17.7)	

Missing data were excluded from percentage calculation

Refused and don’t know data were included in percentage calculation

IQR = Interquartile range; ZAR = South African Rand (ZAR15 ~ US$1); ART = Antiretroviral therapy; STI = sexually transmitted infections;

Ever had any STI symptoms = any symptoms of abnormal vaginal discharge, burning or pain when passing urine, or presence of any genital ulcers/warts;

HSV-2 = Herpes simplex virus type 2;

### Progress towards UNAIDS HIV treatment indicators and overall viral suppression

Table 3 provides the progress towards the UNAIDS indicators. Of the 219 women who tested positive for HIV, questionnaire data were available for 213. Overall, for the “first 90”, 84.5% (95% CI, 79.0–88.8) (n = 180/213) were aware of their HIV positive status and for the “second 90”, 98.3% (95% CI, 95.1–99.4) (n = 173/176) had initiated ART and for the “third 90”, 95.9% (95% CI, 91.8–98.0) (n = 165/172) had achieved viral suppression. Among women in the ≥ 30 year age group, all three indicators had been achieved. However, among adolescents 14–19 years, the “first 90” was achieved in 69.2% (95% CI, 50.0–83.5), and increased among women 20–24 years (75.9% (95% CI, 63.1–85.4) and those 25–29 years (87.5% (95% CI, 76.4–93.8). The “second 90” of ART initiation among adolescents 14–19 years was 88.9% (95% CI 67.2–96.9) and was 100% among the 20–24 and 25–29 age groups. For the “third 90”, viral suppressions among those who had initiated ART exceeded 90% (Table 3). Among all HIV positive women (n = 219), viral suppression was achieved in 90.8% (95% CI 86.3 -94.0) (198/218), (data excludes one participant with no viral load measurement). The overall viral suppression was lowest among adolescents aged 14–19 years (n = 24/29) at 82.8% (95% CI 65.5–92.4) and in women aged 20–24 years (n = 49/55) at 89.1% (95% CI 78.2–94.9). However, viral suppression increased to 94.7% (95% CI 85.6–98.2) among women aged 25–29 years (n = 54/57) and to 92.2% (95% CI 84.0–96.4) among women ≥ 30 years (n = 71/77) (Table 3).

**Table 3:** Proportion of enrolled pregnant adolescents and women achieving each of the UNAIDS 90–90–90 targets and overall HIV viral suppression in rural KwaZulu-Natal, South Africa

Characteristics	HIV testing and linkage to care cascade						Overall HIV viral suppression at viral load < 400 copies per mL among all HIV positive participants	
	“First 90”		“Second 90”		“Third 90”			
	Knowledge of HIV status		HIV positive and on ART		HIV viral suppression at viral load < 400 copies per mL among participants aware of HIV status and reporting to be on ART			
	n/N	% (95% CI)	n/N	% (95% CI)	n/N	% (95% CI)	n/N	% (95% CI)
Overall	180/213	84.5 (79.0- 88.8)	173/176	98.3 (95.1–99.4)	165/172	95.9 (91.8–98.0)	198/218	90.8 (86.3–94.0)
Socio-demographic characteristics								
Age group in years								
14–19	18/26	69.2 (50.0–83.5)	16/18	88.9 (67.2–96.9)	16/16	100 (80.6–100)	24/29	82.8 (65.5–92.4)
20–24	41/54	75.9 (63.1–85.4)	37/37	100 (90.6–100)	35/37	94.6 (82.3–98.5)	49/55	89.1 (78.2–94.9)
25–29	49/56	87.5 (76.3–93.8)	49/49	100 (92.7–100)	47/49	95.9 (86.3–98.9)	54/57	94.7 (85.6–98.2)
≥ 30	72/77	93.5 (85.7–97.2)	71/72	98.6 (92.5–99.8)	67/70	95.7 (88.1–98.5)	71/77	92.2 (84.0–96.4)
Education levels								
Completed high school	86/109	78.9 (70.3–85.5)	98/110	89.1(81.9–93.6)	81/84	96.4 (90.0–98.8)	98/110	89.1 (83.3–94.9)
Incomplete high school	94/104	90.3 (83.2–94.7)	98/106	92.5(85.8—96.1)	84/88	95.5 (88.9–98.2)	98/106	92.5 (87.4–97.5)
Household income per month								
≤ ZAR 5000	85/96	88.5 (80.6–93.5)	82/83	98.8 (93.5–99.8)	78/81	96.3 (89.7–98.7)	87/95	91.6 (84.3–95.7)
> ZAR 5000	37/46	80.4 (66.8–89.3)	36/36	100 (90.4–100)	34/36	94.4 (81.9–98.5)	44/48	91.7 (80.4–96.7)
Don’t know	57/69	82.6 (72.0–89.8)	54/56	96.4 (87.9–99.0)	52/54	96.3 (87.5–99.0)	63/71	88.7 (79.3–94.2)
Marital status								
Married	26/26	100 (87.1–100)	26/26	100 (87.1–100)	25/25	100 (86.7–100)		
							25/25	100 (86.7–100)
Unmarried	154/187	82.4 (76.3–87.1)	147/150	98.0 (94.3–99.3)	140/147	95.2 (90.5–97.7)	171/191	89.5 (84.4–93.1)
Family characteristics								
Biological mother alive								
Yes	110/129	85.3 (78.1–90.4)	104/107	97.2 (92.1–99.0)	97/103	94.2 (87.9–97.3)	118/132	89.4 (83.0–93.6)
No	68/82	82.9 (73.4–89.5)	67/67	100 (94.6–100)	66/67	98.5 (92.0–99.7)	76/82	92.7(84.9–96.6)
Don’t know	2/2	100 (34.2–100)	2/2	100 (34.2–100)	2/2	100 (34.2–100)	2/2	100 (34.2–100)
Biological father alive								
Yes	67/80	83.8 (74.2–90.3)	62/64	96.9 (89.3–99.1)	58/61	95.1(86.5–98.3)	69/80	86.3 (77.0–92.1)
No	110/130	84.6 (77.4–89.8)	108/109	99.1 (95.0–99.8)	104/108	96.3 (90.9–98.6)	124/133	93.2 (87.6–96.4)
Don’t know	3/3	100 (43.9–100)	3/3	100 (43.9–100)	3/3	100 (43.9–100)	3/3	100 (43.9–100)
Behavioral characteristics								
Age at first sex								
At ≤ 16 years	9/11	81.8 (52.3–94.9)	8/9	88.9 (56.5–98.0)	8/8	100 (67.6–100)	10/12	83.3 (55.2–95.3)
At 17–18 years	21/29	72.4 (54.3–85.3)	19/20	95.0 (76.4–99.1)	18/19	96.4 (91.9–98.5)	24/30	89.3 (80.9–94.3)
At > 18 years	144/167	86.2 (80.2–90.6)	140/141	99.3 (96.1–99.9))	134/139	96.4 (91.9–98.5)	156/167	93.4 (88.6–96.3)
Refused	6/6	100 (61–100)	6/6	100 (61–100)	5/5	83.3 (43.6–97.0)	8/9	88.9 (56.5–98.0)
Total number of lifetime sex partners								
1 partner	17/20	85.0 (64.0–94.8)	17/17	100 (81.6–100)	14/17	82.4 (59.0–93.8)	17/21	81.0 (60.0–92.3)
2 or more partners	144/172	83.7 (77.5 -88.5)	138/140	98.6 (94.9–99.6)	135/138	97.8 (93.8–99.3)	162/175	92.6 (87.7–95.6)
Don’t know/Refused	19/21	90.5 (71.1–97.3)	18/19	94.7 (75.4–99.1)	16/17	94.1 (73.0–99.0)	17/20	85.0 (64.0–94.7)
Total number of current sex partners								
1 partner	171/203	82.2 (78.6–88.6)	165/167	98.8 (95.7–99.7)	157/164	95.7 (91.5–97.9)	188/206	91.3 (86.6–94.4)
2 or more partners	1/1	100 (20.7–100)	1/1	100 (20.7–100)	1/1	100 (20.7–100)	1/1	100 (20.7–100)
Refused	8/9	88.9 (56.6–98.0)	7/8	87.5 (52.9–97.8)	7/7	100 (64.6–100)	7/9	77.8 (50.6–94.8)
Alcohol use								
No	112/134	83.6 (76.4–88.9)	109/111	98.2 (93.7–99.5)	101/108	93.5 (87.2–96.8)	120/135	88.9 (82.5–93.1)
Yes	68/79	86.1 (76.8 -92.0)	64/65	98.5 (91.8–99.7)	64/64	100 (94.3–100)	76/81	93.8 (86.4–97.3)
Drug use								
No	156/184	84.8 (79.4–89.7)	152/154	98.7 (95.4–99.6))	144/151	95.4 (90.7–97.7)	169/186	90.9 (85.9–94.2)
Yes	17/22	77.3 (56.6- 89.9)	15/16	93.8 (71.7–98.9)	15/15	100 (79.6–100)	20/23	87.0 (67.9–95.5)
HIV Testing history								
Last HIV test done								
> 12 months	64/69	92.8 (84.1–96.9)	64/64	100 (94.4–100)	60/63	95.2 (86.9–98.4)	62/68	91.2(82.1–95.9)
≤ 12 months	116/144	80.6 (73.3–86.2)	109/112	97.3 (92.4–99.1)	105/109	96.3 (90.9–98.5)	130/144	90.3(84.3–94.1)
Clinical characteristics								
Pregnancies								
Current only	52/71	73.2(61.9–82.1)	48/50	96.0 (86.5–98.9)	46/48	95.8 (86.0–98.8)	64/73	87.7 (78.2–93.4)
2 or more	127/141	90.1 (84.0 -94.0)	124/125	99.2 (95.6–99.9)	118/123	95.9 (90.8–98.3)	131/142	92.3(86.7–95.6)
The first time that you had sex, was your partner circumcised								
No	113/125	90.4 (84.0–94.4)	109/110	99.1 (95.0–99.8)	102/108	94.4 (88.4–97.4)	114/127	89.8 (83.3–93.9)
Yes	30/41	73.2 (58.1–84.3)	30/30	100 (88.6–100)	30/30	100 (88.6–100)	41/42	97.6 (87.7–99.6)
Don’t know	28/37	75.7 (59.9–86.6)	26/27	96.3 (81.7–99.3)	25/26	96.2 (81.1–99.3)	33/37	89.2 (75.3–95.7)
Ever had any STI symptoms								
Yes	66/78	84.6 (75.0–91.0)	62/64	96.9 (89.3–99.1)	60/62	96.8 (89.0–99.1)	70/78	89.7 (81.0–94.7)
No	114/135	84.4 (77.4–89.6)	111/112	99.1 (95.1–99.8)	105/110	95.5 (89.8–98.0)	126/138	91.3 (85.4–95.0)
HSV-2 antibodies								
Positive	160/189	84.7 (78.8–89.1)	154/156	98.7 (95.4–99.6)	148/153	96.7 (92.6–98.6)	176/192	91.7 (86.9–94.8)
Negative	20/24	83.3 (64.1–93.3)	19/20	95.0 (76.4–99.1)	17/19	89.5 (68.6–97.1)	22/26	84.6 (66.5–93.8)

Missing data were excluded from percentage calculation

Refused and don’t know data were included in percentage calculation

IQR = Interquartile range; ZAR = South African Rand (ZAR15 ~ US$1); ART = Antiretroviral therapy; STI = sexually transmitted infections;

Ever had any STI symptoms = any symptoms of abnormal vaginal discharge, burning or pain when passing urine, or presence of any genital ulcers/warts; HSV-2 = Herpes simplex virus type 2;

### Factors associated with achieving UNAIDS indicators and overall HIV viral suppression

Table 4 shows the PRR for factors associated with achieving UNAIDS indicators and overall HIV viral suppression. Women 25 years and older compared to women 14–24 years of age [PRR = 1.22 (95% CI, 1.05–1.42)]; married women compared to unmarried women [PRR = 1.15 (95% CI, 1.06–1.25)] and women with incomplete high school education compared to women who had completed high school education [PRR = 1.16 (95% CI, 1.04–1.30)] were all more likely to achieve the “first 90” indicator. We found no discernable factors associated with achieving the “second 90” indicator of ART initiation, whilst married women compared to those unmarried were more likely to achieve the “third 90” indicator of HIV viral suppression [PRR = 1.11 (95% CI, 1.05–1.18)] (Table 4).

**Table 4:** Baseline characteristics associated with achieving each of the UNAIDS 90–90-90 targets and overall HIV viral suppression among enrolled pregnant adolescents and women in a rural KwaZulu-Natal, South Africa

Characteristics	HIV testing and linkage to care cascade						Overall HIV viral suppression at viral load < 400 copies per mL among all HIV positive participants	
	“First 90”		“Second 90”		“Third 90”			
	Knowledge of HIV status		On ART		HIV viral suppression at viral load < 400 copies per mL among participants aware of HIV status and reporting to be on ART			
	PRR (95% CI)	*P* value	PRR (95% CI)	*P* value	PRR (95% CI)	*P* value	PRR (95% CI)	*P* value
Age group in years								
14–24	Ref		Ref		Ref		Ref	
≥ 25	1.22 (1.05–1.42)	0.01**	1.04 (0.97–1.11)	0.30	0.95 (0.90–1.02)	0.14	1.05 (0.95–1.16)	0.37
Marital Status								
Unmarried	Ref		Ref		Ref		Ref	
Married	1.15 (1.06–1.25)	< 0.001**	1.01 (0.99–1.03)	0.25	1.06 (1.01–1.12)	0.02**	1.11 (1.05–1.18)	< 0.001**
Education levels								
Completed high school	Ref		Ref		Ref		Ref	
Incomplete high school	1.16 (1.04–1.30)	0.01**	1.00 (0.96–1.04)	0.83	0.98 (0.92–1.05)	0.54	1.04 (0.95–1.13)	0.39
Total number of lifetime sex partners								
1 partner	Ref		Ref		Ref		Ref	
2 or more partners	1.12 (0.91–1.37)	0.28	1.03 (0.99–1.07)	0.16	0.82 (0.66–1.03)	0.09	0.89 (0.71–1.11)	0.32

ART = Antiretroviral therapy, PRR = Prevalence Risk Ratio, CI = Confidence Interval, Ref = Reference category

Variables not included in the adjusted model because of collinearity were employment status, ever tested for HIV, ever had sex, age at first sex, lifetime number of sex partners, current number of sex partners

** Significant at *P* value < 0.05

## Discussion

Viral suppression among all pregnant adolescents and women living with HIV attending public sector PHC clinics in rural KwaZulu Natal was 90.8%. This finding is important as it reflects South Africa’s commitment and efforts in scaling up of ART towards reaching the UNAIDS target of 90% of viral suppression towards attaining HIV epidemic control [1, 7]. Despite facing many challenges, the South African HIV treatment program has reached more than 5 of the 7.5 million adults (aged 15 years and older) who are receiving ART especially in a country that has the largest global burden of HIV. However, there remains an urgency that the remaining 2.5 million PLHIV initiate ART and simultaneously those on ART are sustained in care. In parallel it is critically important to monitor whether the increasing ART coverage has the desired effect of reducing HIV incidence towards the target of 1.00 per 1000 person-years [4]. Whilst our study focused on pregnant adolescents and women achieving viral suppression, we expect coverage of ART to be similar even in the general population.

Linked to HIV viral suppression, it is crucial to achieve the “first 90” which requires knowledge of HIV status, and failure to reach this target across key age groups is a major barrier to HIV diagnosis and care continuum. The lower proportion of adolescents and women below the age of 30 years in knowing their HIV positive status ranging from 69.2 to 87.5% is concerning, despite the offer of HIV testing services to all pregnant women accessing the PHC clinics. Therefore, every opportunity must be made to facilitate HIV testing to improve knowledge of HIV status. Although universal HIV testing for pregnant women is available through PHC clinics, the delivery of programs through facilities may create a gap as services may not reach all age groups. Whilst decentralization of services provides easier access to services, new modalities of HIV testing including self -testing [24] may have not reached saturation in some communities. Furthermore, many individuals in these communities face structural and individual level barriers that decrease access to both information about services and to the services themselves, and may also include fear of stigma and discrimination, all of which pose major obstacles to HIV testing [34–36]. Many individuals may also be unaware of the benefits and services to which they are entitled to and often lack skills required to engage with authorities around services. Thus, missed opportunities continue to prevail that impede the benefits of knowledge of HIV status and progress to achieving the “first 90” target.

Towards achieving the “second 90” target, our data show remarkable progress on the uptake of ART among those who were aware of their HIV positive status, however, young adolescents in the 14–19 year age group were least likely to achieve this target (88.9%). Whilst there are concerns that women initiating early ART during pregnancy are more likely to become disengaged over time compared to women starting ART because of advanced HIV disease [37], our study showed that in women who had initiated ART, viral suppression was achieved, showing that women were retained in programs through the supportive environment of health services and family. Adolescent friendly interventions, tailored towards addressing adolescent general and reproductive health needs are therefore a priority to improve HIV testing and linkage to care services [14, 15]. Over the years, suboptimal response to HIV services has been shown and continues to pose a threat in adolescents—a double threat in pregnant adolescents—as they pose high risk of HIV transmission to the unborn baby because of late presentation to health facilities.

The “third 90” target of achieving viral suppression is a critical component towards the goal to epidemic control. It is noteworthy that as per UNAIDS targets, 95.9% of adolescents and women in the study who had knowledge of their HIV status and had initiated ART were virally suppressed, whilst overall 90.8% of all pregnant women living with HIV had achieved viral suppression. The high rates of viral suppression among women on ART, though self-reported, suggest that the health systems have established processes and procedure to ensure HIV care continuum and expelling the notion of the potential to be disengaged. Viral suppression is critical for the PMTCT of HIV, improving mothers’ health outcomes and simultaneously preventing transmission to sex partners [11]. Nevertheless, supporting women during and post-pregnancy to sustain adherence to ART is crucial to maintaining viral suppression in the long term.

Younger women were less likely to have knowledge of their HIV status, directly affecting ART initiation and to achieve viral suppression. Several surveys have demonstrated the challenges in reaching out to key groups of younger individuals [38]; and district and national level population-based studies have shown variable and sub-optimal achievements towards the UNAIDS set targets even at viral load suppression threshold at < 1000 copies per mL [9, 16, 35, 36]. However, our findings show that these targets could be achieved among select populations of pregnant women who do better than women from the general population despite the expansion of HIV related services [21, 37]. Thus, our findings and those of the community-based studies emphasize the need for a targeted approach to reach key groups of individuals, specifically those in the younger age groups. Whilst viral suppression was close to 90%, the gap to reaching 90% was also in the age groups that has the highest HIV incidence rates [39], This trend among young women are similar to the findings from the national antenatal survey which showed the gap in the awareness of HIV positive status and linkage to treatment among HIV positive pregnant women are in the 15 to 24 year age group [40].

Whilst we found no distinct factors associated with viral suppression, married women were more likely to be virally suppressed, highlighting a supportive environment that married women might be in and disclosure of HIV positive status to partners may play an important role in facilitating adherence to ART [41]. Though women 25 years and older, married and those with incomplete high school education were more likely to know their HIV status and linked to HIV care continuum. A major concern is the unacceptably high HIV prevalence of 40.2% among pregnant women [27] reflecting the substantial long-term care and treatment needs for a relatively young population in this community. More importantly, in the age group 14–19 years, HIV prevalence of 22.1% emphasizes that young girls continue to be highly vulnerable to HIV and infections in this age group provides a reliable approximation of incident HIV infection [16]. Given that HIV viral load is highest during this early stage of infection and the gap in achieving the UNAIDS targets in this age group has the potential to sustain the cycle of HIV transmission [42, 43]. These findings remain deeply concerning as many of these young girls were still in school underscoring the need for expanding effective pregnancy and HIV prevention options to protect young girls from early pregnancy and HIV. The evaluation of the impact of school-based interventions on sexual risk behaviours and sexually transmitted infections among young adolescents found that for 14,426 secondary school learners who participated across nine RCTs in Zimbabwe, South Africa, Tanzania, Zambia, Liberia, Swaziland, and Uganda, declines were observed in the number of pregnancies and improvements in HIV/AIDS related knowledge, normative beliefs, knowledge and self-efficacy in condom use, positive attitudes to HIV testing, with a moderate decline in sexual debut and intimate partner violence but no long term effect on condom use, or curable STIs, HSV-2 and HIV infections [44]. With the high rate of attrition, it is possible that risk taking behaviours may increase among learners leaving school prematurely and enhancing risk for HIV acquisition, thus explaining the link between incomplete high school school and low knowledge of HIV status. The key strength of this study was our eligibility criteria of enrolling pregnant women as young as 12 years and together with our age-stratified data allowed us to identify key groups that contribute to achieving HIV viral suppression. However, the study presents some limitations. The study sample of pregnant women from a rural setting living under constrained structural and socio-economic conditions with potentially differential exposure to HIV related services reflects the characteristics of pregnant women accessing services in poor communities and limits the generalizability of our findings to this setting. Additional limitations of this study included the substantial proportion of participants refusing to respond to sensitive behavioral questions limiting the association of these factors to accessing HIV related services to facilitate viral suppression. Since this study was cross-sectional in design no causal link could be established between achieving HIV viral suppression and characteristics of pregnant women.

## Conclusions

This study showed that adolescent pregnant women aged 14–19 years were less likely to achieve viral suppression, underscoring the public health implications of sustained risk of HIV transmission through MTCT and to sexual partners. Thus, greater effort and strong social support are essential to improve HIV knowledge of status and care continuum. The overall HIV viral suppression of 90.8% among all HIV positive pregnant women is encouraging, though still far short of the targets to be achieved by 2025. Thus, HIV knowledge of status and care continuum must remain a collective important public health priority to attain viral suppression, reduce transmission risk and achieve the aspirational goal of HIV epidemic control.

## Supplementary Information

**Additional file 1.** **Figure S1:** Recruitment and enrolment of pregnant adolescents and women in a rural district in KwaZulu-Natal, South Africa between December 2016 to March 2017.

## Data Availability

The datasets used and/or analysed during the current study are available from the corresponding author on reasonable request.

## Abbreviations

ANC: Antenatal clinic
ART: Antiretroviral therapy
BREC: Biomedical Research Ethics Committee
CI: Confidence interval
DOH: Department of Health
IQR: Interquartile Range
KZN: KwaZulu-Natal
NSP: South Africa National Strategic Plan for HIV, STIs and TB
MTCT: Mother to child transmission
PDA: Personal digital assistant
PHC: Primary health care
PLHIV: People living with HIV
PMTCT: Prevention of mother-to-child transmission
PRR: Prevalence risk ratio
STI: Sexually Transmitted Infections
UNAIDS: The Joint United Nations Programme on HIV/AIDS
WHO: World Health Organization
ZAR: South African Rand
