# Supplementary material for: Viral suppression among pregnant adolescents and women living with HIV in rural KwaZulu-Natal, South Africa: a cross sectional study to assess progress towards UNAIDS indicators and Implications for HIV Epidemic Control
Source: Reprod Health. 2022 May 12;19:116. doi: 10.1186/s12978-022-01419-5 (PMC9097174; doi:10.1186/s12978-022-01419-5)
Supplement: Supplementary file 1 — Additional file 1. Figure S1: Recruitment and enrolment of pregnant adolescents and women in a rural district in KwaZulu-Natal, South Africa between December 2016 to March 2017. [file 12978_2022_1419_MOESM1_ESM.docx]

**Additional file**

|  |  |  |  | |  |  |
| --- | --- | --- | --- | --- | --- | --- |
|  |  |  | **610**  Pregnant women approached | |  |  |
|  |  |  |  |  |  |  |
|  |  |  |  |  |  |  |
|  | ***Reasons for non-enrolment*** |  |  |  |  |  |
|  | 27 (4.4%) Refused  37 (6.0%) Other reasons |  |  |  |  |  |
|  | 26 (3.9%) did not return |  |  |  |  |  |
|  | 2 (0.3%) sick/heavily pregnant |  |  |  |  |  |
|  | 5 (0.8%) late for work, no transport |  |  |  |  |  |
|  | 1 (0.2%) mentally challenged |  |  |  |  |  |
|  | 1 (0.2%) did not complete procedures |  |  |  |  |  |
|  | 1 (0.2%) no parental consent (<18) |  |  |  |  |  |
|  | 1 (0.2%) reason not recorded |  |  |  |  |  |
|  |  |  |  |  |  |  |
|  |  |  | **546**  enrolled | |  |  |
|  |  |  |  |  |  |  |
|  |  |  |  |  |  |  |
|  | ***Reasons for excluding in the analysis*** |  |  |  |  |  |
|  | 1 difficult sample collection |  |  |  |  |  |
|  |  |  | **545**  Analysed | |  |  |
|  |  |  |  |  |  |  |
|  | ***545 had blood samples collected*** |  |  |  |  |  |
|  | Analysed for overall HIV prevalence |  |  |  |  |  |
|  | ***539 had completed questionnaires*** |  |  | |  |  |
|  | Analysed for questionnaire data  (6 had incomplete questionnaires) |  |  | |  |  |
|  |  |  |  | |  |  |
|  |  |  |  | |  |  |

***Figure S1:*** Recruitment and enrolment of pregnant adolescents and women in a rural district in KwaZulu-Natal, South Africa between December 2016 to March 2017.
